# Evaluation of 3D‐Printed Polylactic Acid as a Bone Substitute: An Animal Study in a Rat Model

**DOI:** 10.1002/cre2.70201

**Published:** 2025-08-05

**Authors:** Velayudhan Ashok, Mohanraj Karthik Ganesh, Subhabrata Maiti, Deepak Nallaswamy, Artak Heboyan

**Affiliations:** ^1^ Department of Prosthodontics and Implantology, Saveetha Dental College and Hospitals, Saveetha Institute of Medical and Technical Sciences Saveetha University Chennai Tamilnadu India; ^2^ Biomedical Research Unit and Lab Animal Centre (BRULAC), Saveetha Dental College and Hospitals, Saveetha Institute of Medical and Technical Sciences Saveetha University Chennai Tamilnadu India; ^3^ Department of Prosthodontics, Faculty of Stomatology Yerevan State Medical University after Mkhitar Heratsi Yerevan Armenia; ^4^ Department of Prosthodontics, School of Dentistry Tehran University of Medical Sciences Tehran Iran

**Keywords:** animal study, bone regeneration, polylactic acid, scaffolds

## Abstract

**Objectives:**

Bone repair and regeneration are important processes for treating bone defects and injuries. However, traditional bone grafts like autografts and allografts have limitations, such as complications at the donor site and immune rejection. As a result, there is growing interest in using polylactic acid (PLA), a biodegradable and biocompatible material, as a synthetic bone substitute. This study aims to evaluate the effectiveness of 3D‐printed PLA scaffolds as bone substitutes using a rat model.

**Material and Methods:**

PLA scaffolds with dimensions of 2 × 2 × 4 mm and 2 × 2 × 8 mm were fabricated using the CUBEX‐TRIO 3D printer. Twelve male Wistar rats were divided into four groups based on defect size (4 and 8 mm) and observation period (4 weeks and 8 weeks). The surgical procedures involved creating discontinuity defects in the rats' zygoma and implanting PLA scaffolds that were stabilized with a bio‐membrane. Bone regeneration was assessed through radiographic analysis and histological examination.

**Results:**

Radiographic analysis confirmed the formation of bone in the grafted regions. Histological analysis revealed connective tissue formation at the defect edges and scaffold surface at both 4 and 8 weeks. In the 4 mm defect group, the transformation of connective tissue into chondrocytes and endochondral ossification was observed at 8 weeks, indicating successful bone regeneration. However, in the 8 mm defect group, bone formation was not as evident, suggesting limitations in the osteoinductive potential of PLA scaffolds for larger defects.

**Conclusions:**

The 3D‐printed PLA scaffolds show promise as bone substitutes for small to moderate‐sized defects due to their effective biocompatibility and osteoinductive potential. Further studies are needed to optimize their performance for larger defects, potentially enhancing their clinical application in bone repair and regeneration.

## 1 Introduction

Bone repair is one of the most common regeneration procedures done in our human body. Bone regeneration is done using bone grafts. Bone substitutes are essential in medical applications, particularly for treating bone defects and injuries. These materials act as scaffolds to support the growth of new bone, facilitating the repair and regeneration of damaged tissues. The types of bone grafts used are autogenous, allografts, xenografts, and alloplastic. The autogenous bone grafts are considered the gold standard since the grafts are taken from the same person (Sakkas et al. 2017). The autogenous bone grafts have excellent osteoinduction, osteoconduction, and osteogenesis properties (Fillingham and Jacobs 2016). Traditional methods, such as autografts and allografts, have limitations like donor site complications and potential immune rejection. Postsurgical complications and pain in donor areas are the main drawbacks while utilizing autogenous bone grafts (Nkenke and Neukam 2014). Allografts have good osteoconduction properties but reduced osteoinduction properties (Brydone et al. 2010). Transmission of disease and inferior healing are reported in allografts compared with autogenous bone grafts (Ehrler and Vaccaro 2000). The usage of alloplastic grafts is emerging in a rapid phase in recent years due to the increased availability of the material. The most common alloplastic materials are hydroxyapatite, calcium phosphate, Bioglass, and carbon nanotubes (Ghanaati et al. 2010). Recently, resin‐based grafts have been introduced in the field of bone regeneration (Neovius and Engstrand 2010). Resin‐based bone grafts have better advantages like good mechanical properties, processability, and porousness (Kuperkar et al. 2024).

Large bone defects from pathologies like lateral periodontal cysts challenge dental rehabilitation by compromising structure, aesthetics, and function. Guided bone regeneration (GBR) is a proven technique, employing barrier membranes to promote osteogenesis while excluding non‐osteogenic cells. Combined with grafts (e.g., autografts, allografts, or biomaterials), GBR enhances outcomes. GBR treatment of a lateral periodontal cyst achieved significant bone regeneration. Emerging technologies, including bioactive scaffolds, BMPs, and stem cell therapy, further enhance osteogenesis and healing. GBR, paired with advanced biomaterials and regenerative approaches, ensures effective management of large bone defects with predictable results (Ramalingam et al. 2019). Platelet‐Rich Fibrin (PRF) is a biocompatible, autologous material derived from a patient's blood through centrifugation. It acts as a natural scaffold rich in growth factors like platelet‐derived growth factor (PDGF) and transforming growth factor‐beta (TGF‐β), promoting wound healing, angiogenesis, and bone regeneration. When combined with other osteoinductive adjuncts such as bone morphogenetic proteins (BMPs), demineralized bone matrix (DBM), or nano‐hydroxyapatite, PRF enhances osteogenesis and accelerates bone remodeling. These adjuncts synergistically provide a favorable microenvironment for stem cell proliferation and differentiation, optimizing outcomes in dental implantology, maxillofacial surgeries, and periodontal regeneration. This combination bridges the gap between biology and clinical applications (Pavlovic et al. 2021). Synthetic bone substitutes have emerged as a promising alternative, designed to address these issues. They need to be biocompatible, support cell attachment and growth, and have mechanical properties similar to natural bone. Advances in materials science have led to a range of synthetic bone substitutes tailored for specific medical needs. Polylactic acid is one of the resin‐based grafts that are used in bone regeneration. Polylactic acid grafts have excellent biocompatible and degradable properties (Danhier et al. 2012). Polylactic acid degrades by hydrolysis method. The water molecule breaks down the ester bond, forming the degradation. If the poly is combined with growth factors, it promotes effective bone regeneration.

3D printing technology has transformed the production of customized medical implants, including bone substitutes. This technology allows for the precise creation of complex structures that closely replicate the intricate architecture of natural bone. Polylactic acid (PLA) is a notable material in 3D printing due to its biodegradability, biocompatibility, and suitable mechanical properties. PLA is a thermoplastic made from renewable resources such as corn starch or sugarcane, making it environmentally friendly. Its degradation into lactic acid, a substance naturally found in the body, enhances its suitability for biomedical use. The objective of this study is to evaluate the effectiveness of 3D‐printed PLA as a bone substitute using a rat model, with the aim of assessing its potential for clinical application in bone repair and regeneration.

## 2 Materials and Methods

### 2.1 Fabrication of Polylactic Acid Bone Scaffold

The scaffold is created using the CUBEX 3D sculpt software, with dimensions of 2 × 2 × 4 mm and 2 × 2 × 8 mm (W × H× L). The CUBEX file is then transferred via USB to the CUBEX‐TRIO 3D printer (USA), which utilizes plastic jet printing technology. Before printing, the print pad is coated with CUBEX glue. Once the printing process is complete, the support materials are trimmed and smoothed.

### 2.2 Sample Size Calculation and Animal Selection

The sample size for each group was determined to be 6, calculated using GPower (Version 3.0.10). The level of significance was set at 0.05, with a study power of 0.80. Male Wistar rats, weighing between 280 and 300 g and randomly bred at the Biomedical Research Unit and Laboratory Animal Centre (BRULAC), Saveetha Institute of Medical and Technical Sciences, Chennai, India, were used for the study. The rats were housed in polypropylene cages and kept at a temperature of 25 ± 2°C with a natural light/dark cycle. They were provided with laboratory animal feed and filtered water. The study commenced following approval from the Institutional Animal Ethical Committee (BRULAC/SDCH/SIMATS/IAEC/3‐2020/054) of Saveetha Institute of Medical and Technical Sciences, Chennai, India.

### 2.3 Experimental Groups

Twelve rats were divided into four groups of three rats each.

Group I: A discontinuity defect of 4 mm was made in the zygoma of rat, and a polylactic acid scaffold in the dimension of 2 ×2 × 4 mm was inserted and left for 4 weeks. The scaffold was stabilized with bio‐membrane.

Group‐II: A discontinuity defect of 4 mm was made in the zygoma of rat, and a polylactic acid scaffold in the dimension of 2 × 2 × 4 mm was inserted and left for 8 weeks. The scaffold was stabilized with bio‐membrane.

Group‐III: A discontinuity defect of 8 mm was made in the zygoma of rat, and a polylactic acid scaffold in the dimension of 2 × 2 × 8 mm was inserted and left for 4 weeks. The scaffold was stabilized with bio‐membrane.

Group‐IV: A discontinuity defect of 8 mm was made in the zygoma of rat, and a polylactic acid scaffold in the dimension of 2 × 2 ×8 mm was inserted and left for 8 weeks. The scaffold was stabilized with bio‐membrane.

### 2.4 Surgical Procedure

**Figure 1:**
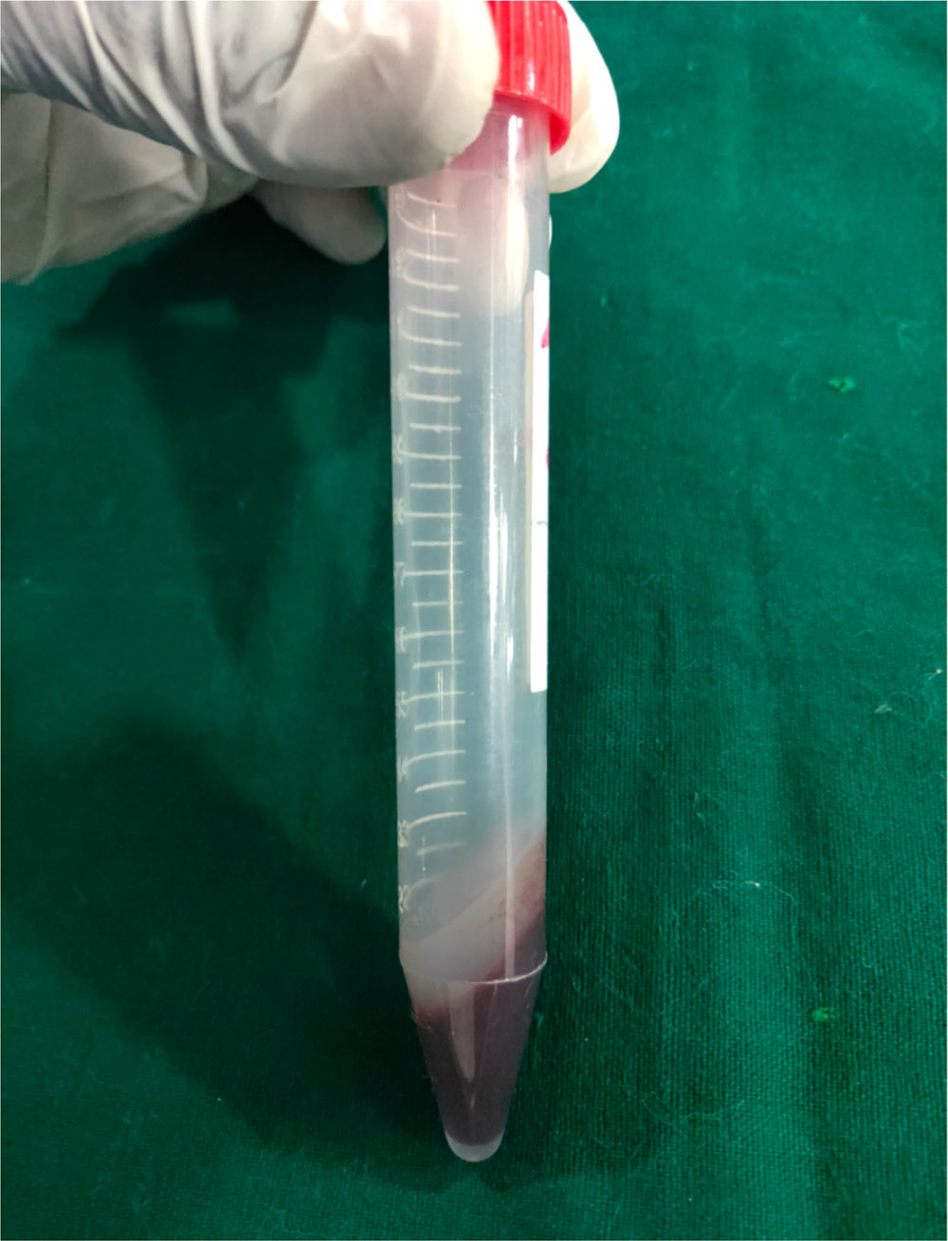
PRF obtained after centrifugation of the collected blood.

**Figure 2:**
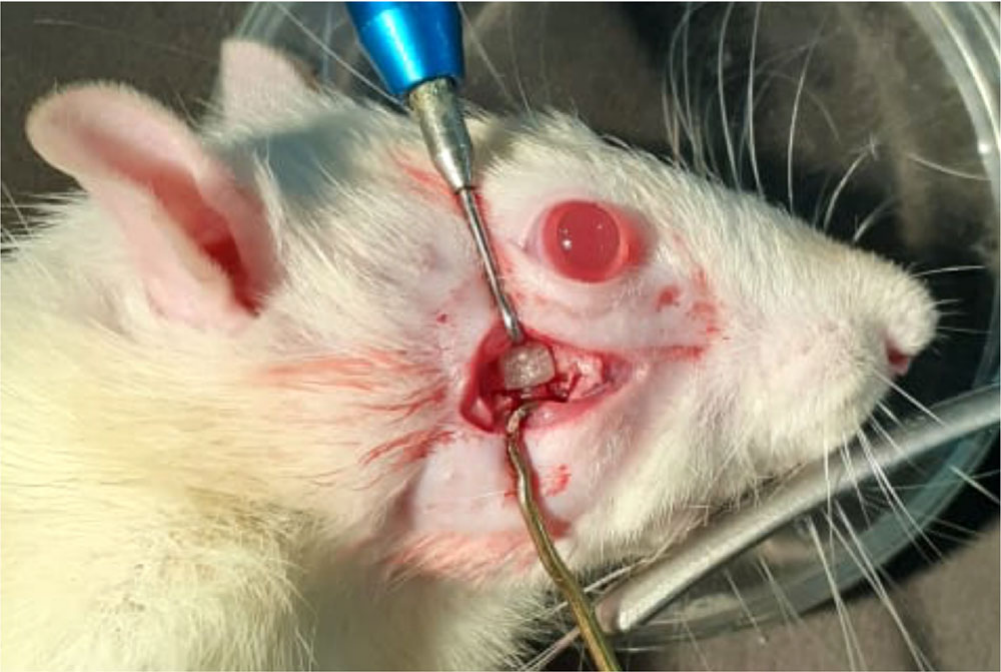
PLA graft placed after creating discontinuity defect in the zygoma.

**Figure 3:**
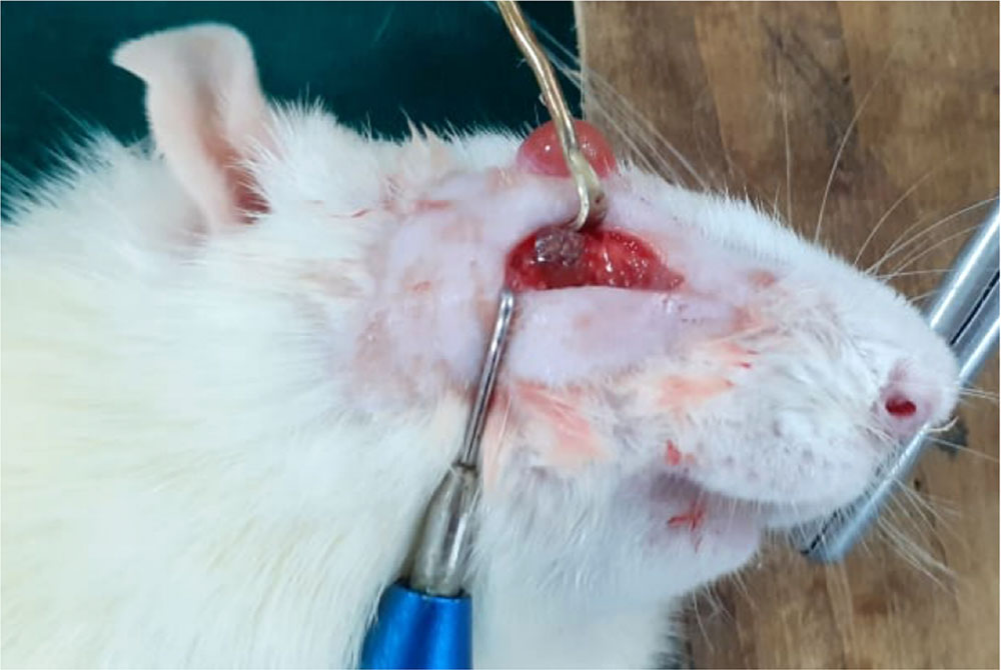
Barrier biomembrane wrapped around the graft and zygoma bone for stabilization of the graft.

Surgical procedures were conducted in a meticulously sterile environment within an animal laboratory surgical room. Rats were appropriately anesthetized using ketamine hydrochloride administered intraperitoneally (ip) at a dosage of 70 mg/kg body weight, supplemented with xylazine administered intramuscularly (im) at a dosage of 10 mg/kg body weight. For blood sample collection, the periorbital plexus was accessed by carefully inserting a capillary tube into the medial canthus of the eye. Subsequently, the collected blood was subjected to centrifugation to isolate Platelet‐Rich Fibrin (PRF). This process allows for the extraction of PRF fibrin, which holds promise for various medical applications due to its regenerative properties (Figure 1). PRF has been recognized for its role in enhancing bone regeneration. To facilitate this process, the lateral surface of the face, particularly around the zygomatic arch, was prepared by shaving and aseptically cleaning with a betadine solution. A precise 2 cm horizontal skin incision was made on the lateral aspect of the face, revealing the underlying fascia and muscles. These tissues were gently retracted to expose the zygomatic arch clearly. Subsequently, the central portion of the zygomatic arch was meticulously cleared of any connective tissue and muscle attachments within a 5 mm radius. A through‐and‐through osseous defect, spanning approximately 4 mm, was meticulously created on one side of the jaw using a trephine bur mounted on a straight handpiece driller under precise motor regulator control. Throughout the drilling process, periodic irrigation with saline water ensured tissue preservation. Special care was taken during the surgical procedure to avoid damaging blood vessels. Poly Lactic Acid material blocks, each measuring 2 × 2 × 4 mm, were then securely implanted into the prepared site within the zygomatic arch. The aim was to ensure snug lodging of the implant within the bone defect area to support optimal bone regeneration (Figure 2). The implanted PLA block in the zygomatic arch was wrapped with PRF for better healing and absorbable algae‐based hydrogel layer to promote blood stay and to make the implant immovable (Figure 3). In another experimental group, the identical procedure was performed with an 8 mm length osseous defect, and PLA blocks measuring 2 × 2 × 8 mm were placed within the bony defect. Following this, tissue flaps were sutured using resorbable Vicryl 5/0 suture threads from Ethicon, Somerville, NJ, USA, and betadine ointment was applied to the sutured area. Subsequently, the rats were housed individually in separate cages to prevent interference with the surgical sites. To alleviate postoperative discomfort, Fevastin at a dosage of 10 mg/kg body weight (administered intramuscularly) and Diclofenac at a dosage of 10 mg/kg body weight (administered orally) were provided periodically. Daily examinations were conducted to monitor the surgical sites for any signs of inflammation or infection, ensuring the well‐being of the rats throughout the recovery period.

### 2.5 Radiograph

A lateral view of the skull radiograph was taken to verify the bone formation in the grafted region.

### 2.6 Histological Preparation

Tissue samples were collected at the end of 4 weeks and 8 weeks by euthanizing the animals in a CO_2_ chamber, and the zygomatic arch bone containing the PLA implant was cut and the required area alone was dissected, photographed, and processed for histopathological examination. The zygomatic bony part with the implant was fixed in 10% neutral buffered formalin and processed for the decalcification process for the routine histopathological procedure.

The fixed tissues were taken out and later decalcified in 20% formic acid for 7 days. Afterward, the samples were embedded in paraffin and serial sections were cut at a thickness of 5 µm. The sections were then processed for Hematoxylin & Eosin staining and mounted permanently in DPX. The stained tissue sections were observed under a microscope and photographed at 400× magnification. The histopathological alterations were examined and analyzed thoroughly.

## 3 Results

### 3.1 Radiographic Analysis

**Figure 4:**
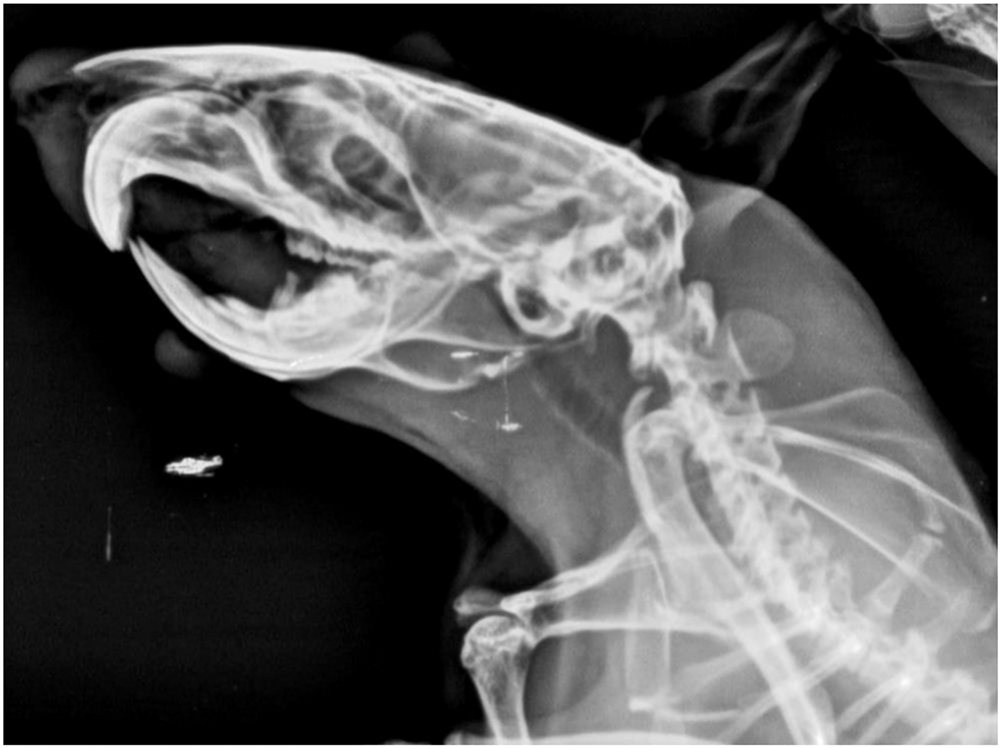
Lateral view of the skull to verify the bone formation in the defect area.

A lateral view skull X‐ray was obtained to confirm the efficacy of bone formation within the grafted region. This imaging technique allowed for the visualization and assessment of the newly formed bone in the area where the graft material was implanted. By capturing a lateral perspective of the skull, the X‐ray provided detailed insight into the extent and quality of bone regeneration following the surgical procedure (Figure 4).

### 3.2 Histological Analysis

**Figure 5:**
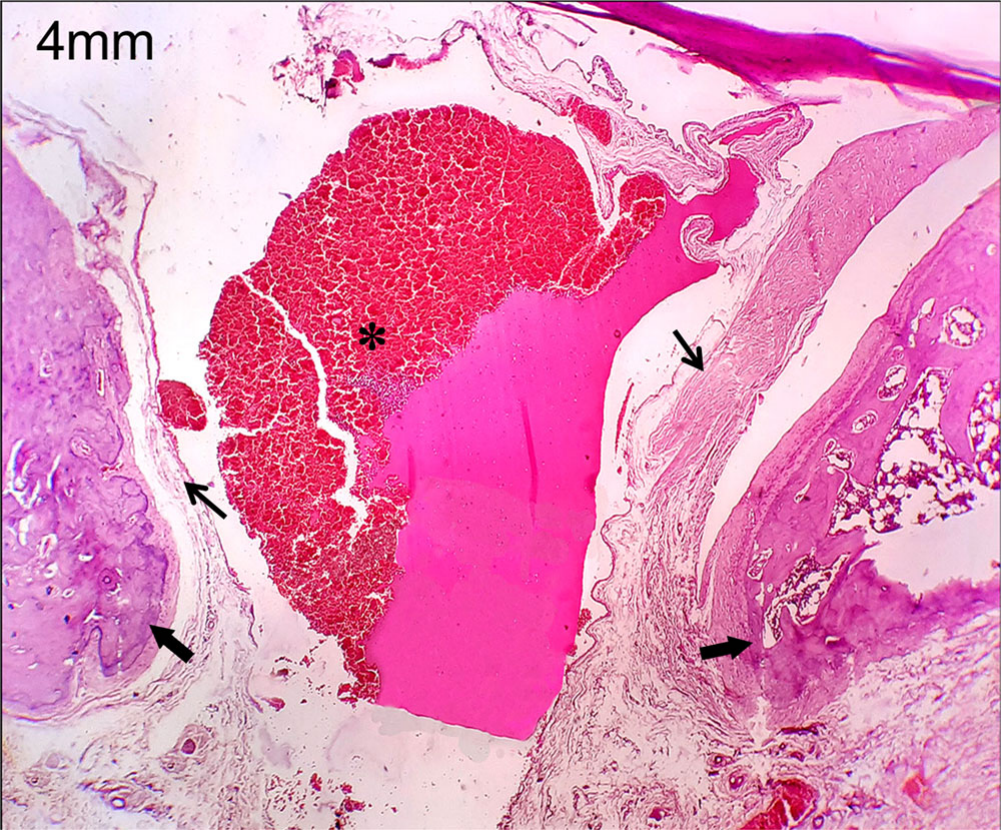
Histological picture after 4 weeks of 4 mm discontinuity defect replaced with poly lactic acid graft, showing connective tissue formation on the edges of the zygomatic bone and over the graft. Asterisk—PLA material with blood cells; thick arrows—bone tissue; thin arrows—connective tissue fibers.

**Figure 6:**
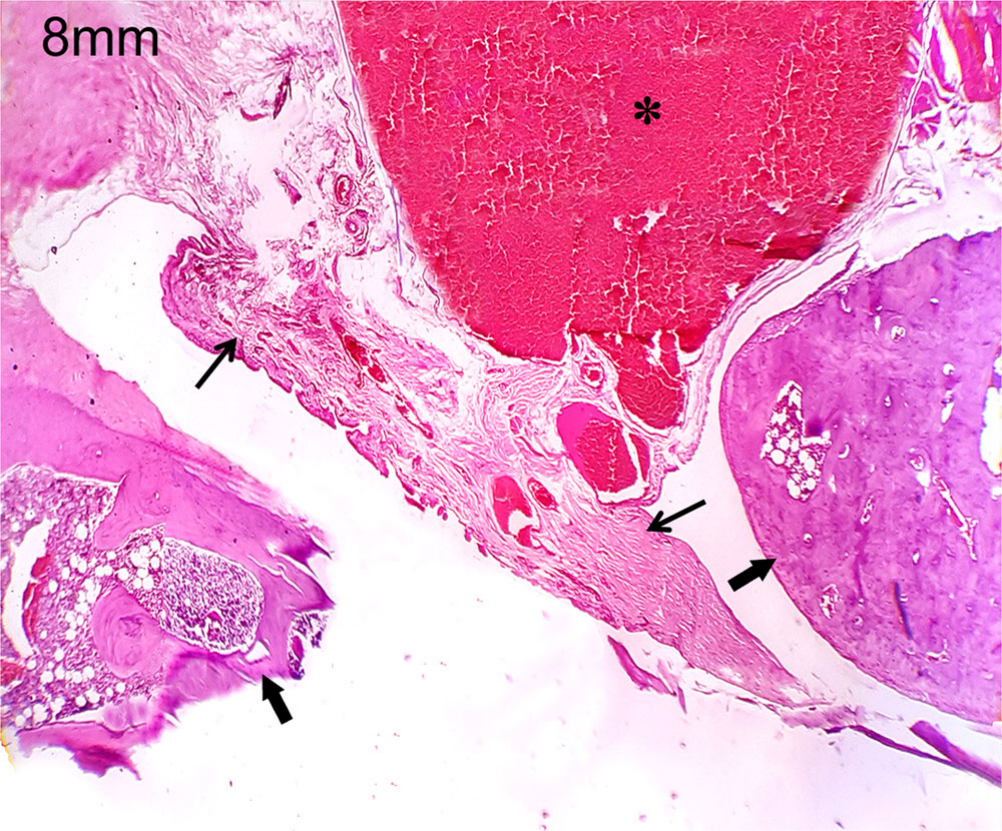
Histological picture after 4 weeks of 8 mm discontinuity defect replaced with poly lactic acid graft, showing connective tissue formation on the edges of the zygomatic bone and over the graft. Asterisk—PLA material with blood cells; thick arrows—bone tissue; thin arrows—connective tissue fibers.

**Figure 7:**
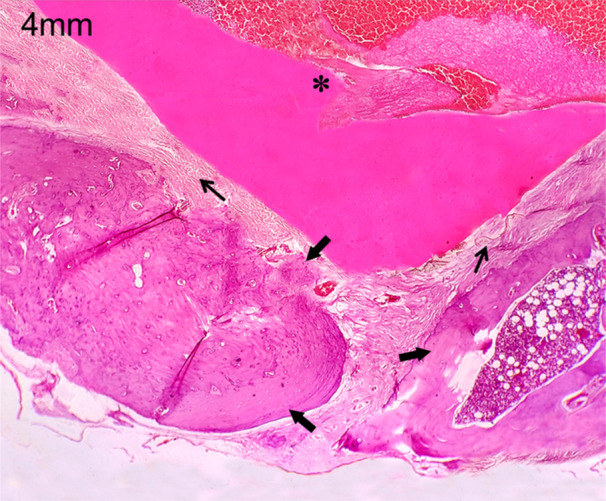
Histological picture after 8 weeks of 4 mm discontinuity defect replaced with poly lactic acid graft, showing transformation of connective tissue into chondrocytes. Asterisk—PLA material with blood cells; thick arrows—bone tissue; thin arrows—connective tissue fibers.

**Figure 8:**
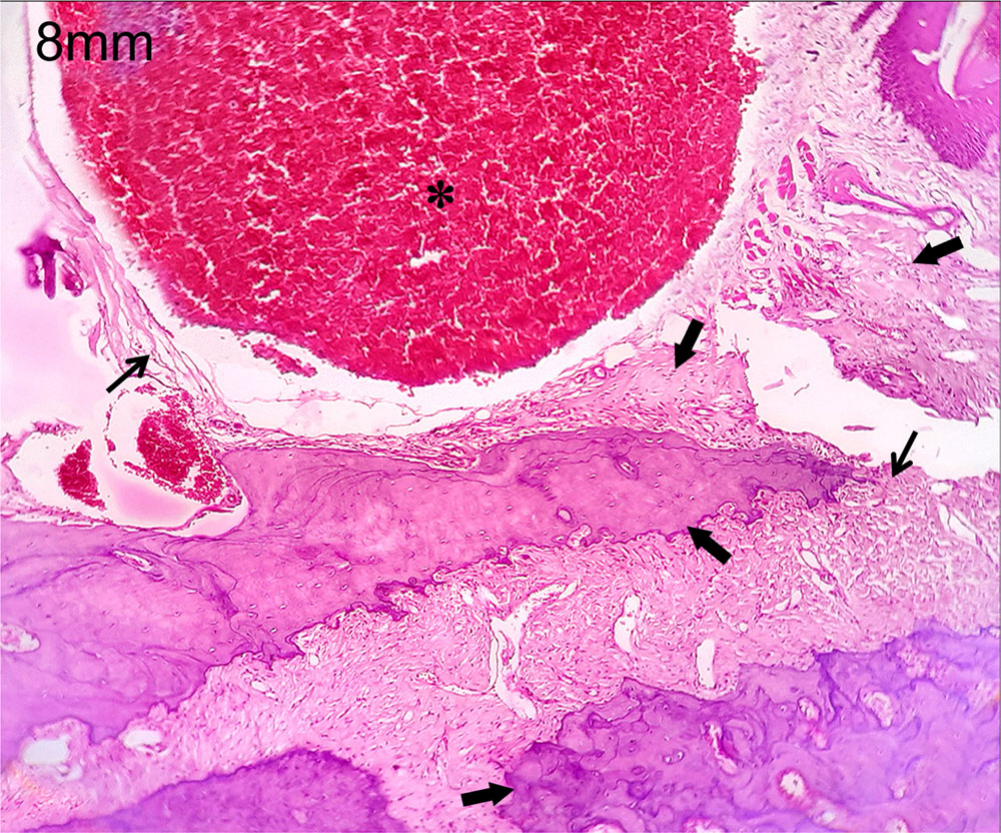
Histological picture after 8 weeks of 8 mm discontinuity defect replaced with poly lactic acid graft. Histological picture shows no transformation in the connective tissue. Asterisk—PLA material with blood cells; thick arrows—bone tissue; thin arrows—connective tissue fibers.

In this study, the polylactic acid bone scaffold was implanted into the discontinuity defect of the zygoma rat to evaluate bone regeneration. As the bone volume is sufficient enough for tissue growth, the connective tissue formation was seen at the two edges of the zygomatic bone and also on the surface of polylactic acid, which is in the defect site (Figure 5). This characteristic histopathological feature is seen in both the 4 and 8 mm discontinuous defects at 4‐week time period (Figure 6). At 8‐week time period, connective tissue formation was seen in both the 4 and 8 mm discontinuous defects, but in the 4 mm discontinuous defect group, the transformation of connective tissue into chondrocytes led to endochondral ossification, leading to bone callus formation (Figure 7). This could be due to the osteoinductive and osteoconductive properties of polylactic acid material at 4 mm discontinuous defect size than at 8 mm discontinuous defect size. In the 8 mm discontinuous defect, the bone formation was not obvious in this group as the distance may be too long for the bones to initiate osteoinductive behavior and thus fail to transform connective tissues into bone cells and be unable to influence bone formation (Figure 8). Since the polylactic acid material was made porous to establish blood flow, the blood cells and inflammatory cells were seen lodging at the defect site. This facilitates the healing capacity of the bony defect, inducing osteogenesis at this critical size defect model of the zygomatic bone.

## 4 Discussion

Bone grafts are the second most common transplantation material done, after blood transfusion, in the human body. Due to the large demand, various artificial bone substitutes are being tried. In alloplastic bone grafts, hydroxyapatite and tricalcium phosphate are most commonly used. Resin‐based bone grafts are widely used due to their many good properties. Resin‐based bone grafts can be manufactured using additive technology. Polylactic acid bone grafts are popular due to their better biocompatible, biodegradable, and mechanical properties. Polylactic bone scaffolds can be customized based on necessity. The biodegradation of the polylactic acid depends upon the composition (Wu et al. 2023; de Bomfim et al. 2023). The results indicate that the 3D‐printed PLA scaffolds exhibited excellent biocompatibility, as evidenced by the absence of adverse inflammatory reactions in the implantation sites. The histological analysis demonstrated connective tissue formation at the edges of the zygomatic bone and on the surface of the PLA scaffold within the defect site at both 4‐ and 8‐week intervals. This suggests that the PLA scaffolds were well‐tolerated by the host tissue and provided a suitable environment for tissue integration and healing. The PLA scaffolds showed significant osteoconductive properties, particularly in the 4 mm defect groups. At the 8‐week mark, the transformation of connective tissue into chondrocytes, leading to endochondral ossification and subsequent bone callus formation, was observed in the 4 mm defect group. This indicates that the PLA scaffolds effectively facilitated bone regeneration through osteoconduction, supporting new bone growth along the scaffold structure. The porous nature of the PLA scaffolds likely contributed to this process by allowing vascularization and the infiltration of osteogenic cells. An emerging focus in bone regeneration is the use of platelet‐derived or biological adjuncts to enhance scaffold osteoinductivity. Platelet‐rich fibrin (PRF), known for releasing growth factors like Platelet‐Derived Growth Factor (PDGF), Vascular Endothelial Growth Factor (VEGF), and Transforming Growth Factor‐beta (TGF‐β), supports cellular migration, angiogenesis, and osteogenesis. Studies, such as Alkindi et al. (2021) demonstrate the synergistic potential of PDGF with osteoconductive scaffolds in segmental bone defect repair. PRF and PDGF may amplify scaffold integration and signaling pathways for bone formation (Alkindi et al. 2021).

More the lactic acid, the faster the degradation (Tejedor‐Sanz et al. 2022; Whulanza et al. 2021; Shenoy et al. 2023). Depending on the necessity of degradation, the composition of the polylactic acid is selected for use. The pure form polylactic acid has lesser degradation properties than with the combination of different graft materials (Ranakoti et al. 2022). The two‐layered polylactic acid scaffold has been tried by Vivo, and it has better degradation properties than the single layer. In two‐layered polylactic acid, two scaffolds with different densities have been created and two layers were sandwiched (Schmiedova et al. 2021; Wang et al. 2022). More the porous the bone grafts, the better the osteoconductivity (Bembi et al. 2016; Aldemir Dikici et al. 2023). The Cubex 3D printer has four modes of density. Hollow: The part will have no internal structure. Thin: The part will have a small amount of internal structure to give it support. Medium: The part will have a good internal structure supporting it. Heavy: A heavy part will have a strong internal structure (Konka et al. 2021). While the 4 mm defects showed promising bone regeneration, the 8 mm defects did not exhibit similar outcomes. The lack of obvious bone formation in the 8 mm defect group suggests that the critical size of the defect plays a significant role in the effectiveness of the PLA scaffolds. The distance may be too great for the scaffold to sufficiently support the transformation of connective tissues into bone cells (Zhao et al. 2021). This indicates a limitation in the osteoinductive potential of PLA scaffolds when used in larger defects, which may require additional factors or modifications to enhance their effectiveness in promoting bone formation over greater distances.

The zygomatic arch is an immovable bone and needs less strength than a movable bone. In this study, a thin density bone scaffold was made. Thin‐density bone scaffold degrades more quickly than thick density. Moreover, thin‐density bone scaffolds will be more porous. Bone scaffold with pores aids in blood flow inside the grafts and accumulation of cells that aid in osteogenesis. A combination of polylactic acid with bone marrow–derived mesenchymal stem cells showed better osteogenesis properties than a pure form of poly (Ewers 2005; Fettouh et al. 2023). Based on the manufacturing of polylactic acid, Lee et al. (2012) compared the syringe type and filament type and concluded that the syringe type needed high temperature for processing, resulting in lower molecular weight and leaching of by‐products. In our study, the processing of poly was done with the filament method. This helps in retaining the properties of the polylactic acid. By centrifugation of autogenous blood, platelet‐rich plasma (PRP) can be obtained. This platelet‐rich plasma contains a large number of growth factors and proteins. Adding platelet‐rich plasma over the defect site improves repair. In this study, retro‐orbital blood collection was made for PRP preparation. The PRP is then placed over the defect after the placement of the bone scaffold after better healing. Beane et al. (2014) described that adipose tissue‐derived mesenchymal stem cells, when combined with polylactic acid, have better proliferation ability. The polylactic acid scaffold has hydrophobic properties. This decreases protein and cell adhesion. For better adhesion, extracellular matrix materials like fibrin, fibronectin, and col can be applied over the scaffold. The artificial extracellular matrix material is also used by many researchers for better cell adhesion. In our study, biomembrane has been tried as a stabilization agent. It aids in better adhesion. According to previous literature, to achieve better mechanical properties, a combination of hydroxyapatite (HA) with polylactic acid can be used (Maraveas et al. 2024; Ponnanna et al. 2021). Hydroxyapatite has better biocompatibility and is osteoconductive. But it is brittle, has poor degradability, and lacks osteoinductivity. A combination of polylactic acid with hydroxyapatite provides better bone regeneration. Ghanaati et al. (2010) described the excellent biocompatible property of tricalcium phosphate. However, tricalcium phosphate has poor strength and processability. Combining tricalcium phosphate with polylactic acid gives better healing properties (Cheng et al. 2018).

While PLA scaffolds have been extensively studied for their application in bone regeneration, this study provides unique insights by specifically focusing on: defect size impact. It evaluates the regenerative potential of 3D‐printed PLA scaffolds in two distinct defect sizes (4 mm and 8 mm) within a zygomatic bone model in rats, creating discontinued defects. Most previous studies do not emphasize the critical role of defect size in scaffold performance, which is a key determinant of clinical applicability. By assessing bone healing over 4 and 8 weeks, the study provides a time‐dependent perspective on the progression of connective tissue transformation and endochondral ossification, which is not always explored in detail in earlier studies. The use of a bio‐membrane to stabilize the scaffolds offers a novel approach that may improve scaffold integration and support osteogenesis, a method that has not been widely evaluated in literature focusing on PLA. The successful regeneration observed in the 4 mm defect group highlights the clinical potential of 3D‐printed PLA scaffolds in repairing small‐ to moderate‐sized bone defects, such as facial trauma or minor reconstructive surgeries. The limited osteoinductive potential observed in the 8 mm defect group underscores the necessity to enhance PLA scaffolds for larger defects. This could involve modifications such as bioactive coatings (e.g., hydroxyapatite), growth factor incorporation, or composite scaffold designs that mimic natural bone properties. The study's findings pave the way for the optimization of PLA scaffolds to address larger defects, enhancing their applicability in orthopedic and maxillofacial surgeries.

Future studies should focus on enhancing the osteoinductive properties of PLA scaffolds for larger defects. This could involve the incorporation of growth factors or bioactive molecules to stimulate bone formation and improve scaffold performance. Additionally, exploring the combination of PLA with other biomaterials or advanced fabrication techniques could lead to the development of more effective bone substitutes. Long‐term studies are also needed to assess the degradation rate of PLA scaffolds and their long‐term impact on bone regeneration and remodeling. In the future, 3D‐printed bone scaffolds can be fabricated using the following technique. Computed tomography was exported as an STL file. Mirror imaging is done on the contralateral side of the defect. Using a 3D printer, the bone scaffold is printed before the surgery is performed. During the surgery, the scaffold is implanted in the defect. This technique acquires good aesthetics.

## 5 Conclusion

This study highlights the potential of 3D‐printed PLA scaffolds as effective bone substitutes for small‐ to moderate‐sized defects, demonstrating excellent biocompatibility and promoting endochondral ossification. The findings provide valuable insights into PLA's osteoinductive properties, particularly for small defects, while emphasizing the need for optimization to address challenges in larger defects. Clinically, the use of PLA scaffolds offers a cost‐effective, customizable, and biodegradable alternative to traditional grafts, reducing complications associated with donor‐site morbidity and immune rejection. This study underscores the importance of further studies to enhance scaffold design, mechanical strength, and bioactivity for broader clinical applications in bone regeneration.

## Author Contributions

**Velayudhan Ashok:** conceptualization. **Mohanraj Karthik Ganesh:** methodology. **Subhabrata Maiti:** software. **Artak Heboyan and Velayudhan Ashok:** validation. **Subhabrata Maiti, Mohanraj Karthik Ganesh and Deepak Nallaswamy:** formal analysis. **Velayudhan Ashok and Mohanraj Karthik Ganesh:** investigation. **Mohanraj Karthik Ganesh and Deepak Nallaswamy:** resources. **Velayudhan Ashok:** data curation. **Velayudhan Ashok, and Mohanraj Karthik Ganesh:** writing – original draft preparation. **Subhabrata Maiti and Deepak Nallaswamy:** writing – review and editing. **Artak Heboyan, Deepak Nallaswamy, and Subhabrata Maiti:** visualization. **Subhabrata Maiti and Artak Heboyan:** supervision. **Subhabrata Maiti:** project administration.

## Ethics Statement

The study commenced following the approval from the Institutional Animal Ethical Committee (BRULAC/SDCH/SIMATS/IAEC/3‐2020/054) of Saveetha Institute of Medical and Technical Sciences, Chennai, India.

## Consent

The authors have nothing to report.

## Conflicts of Interest

The authors declare no conflicts of interest.

## Data Availability

The data that support the findings of this study are available from the corresponding author upon reasonable request.
